
JOURNAL INFORMATION
==============================
NLM Title Abbreviation: J Mol Cell Biol
Journal ID: J Mol Cell Biol
NLM Journal ID: 101503669
Journal ID: jmcb

Journal of Molecular Cell Biology

ISSN: 1674-2788
EISSN: 1759-4685

ARTICLE INFORMATION
==============================
PMCID: PMC8690227
PMID: 34850919
DOI: 10.1093/jmcb/mjab075
Article ID: mjab075
Article version: 1
Subjects: Research Advance, AcademicSubjects/SCI01180

Comments on ‘An airway organoid-based screen identifies a role for the HIF1α‒glycolysis axis in SARS-CoV-2 infection’

Duan Xiaohua Department of Surgery, Weill Cornell Medicine, 1300 York Ave., New York, NY 10065, USA
State Key Laboratory of Oncogenes and Related Genes, Center for Single-Cell Omics, School of Public Health, Shanghai Jiao Tong University School of Medicine, Shanghai 200025, China

Wang Hui State Key Laboratory of Oncogenes and Related Genes, Center for Single-Cell Omics, School of Public Health, Shanghai Jiao Tong University School of Medicine, Shanghai 200025, China

Ho David D Aaron Diamond AIDS Research Center, Columbia University Vagelos College of Physicians and Surgeons, New York, NY 10032, USA

Schwartz Robert E Division of Gastroenterology and Hepatology, Department of Medicine, Weill Cornell Medicine, 1300 York Ave., New York, NY 10065, USA
Department of Physiology, Biophysics and Systems Biology, Weill Cornell Medicine, 1300 York Ave., New York, NY 10065, USA

Evans Todd Department of Surgery, Weill Cornell Medicine, 1300 York Ave., New York, NY 10065, USA

https://orcid.org/0000-0002-6294-5187 Chen Shuibing Department of Surgery, Weill Cornell Medicine, 1300 York Ave., New York, NY 10065, USA

Correspondence to: Shuibing Chen, E-mail: shc2034@med.cornell.eduCorrespondence to: Todd Evans, E-mail: tre2003@med.cornell.eduCorrespondence to: David D. Ho, E-mail: dh2994@cumc.columbia.eduCorrespondence to: Robert E. Schwartz, E-mail: res2025@med.cornell.eduCorrespondence to: Hui Wang, E-mail: huiwang@shsmu.edu.cn
Electronic publication date: 2021 Nov 30
Electronic Location ID: mjab075
Copyright: © The Author(s) 2021. Published by Oxford University Press on behalf of Institute of Biochemistry and Cell Biology, Shanghai Institutes for Biological Sciences, Chinese Academy of Sciences.
Copyright year: 2021
License: This is an Open Access article distributed under the terms of the Creative Commons Attribution License (https://creativecommons.org/licenses/by/4.0/), which permits unrestricted reuse, distribution, and reproduction in any medium, provided the original work is properly cited.
License URL: https://creativecommons.org/licenses/by/4.0/

edited-state: accepted-manuscript
article-lifecycle: PAP

==============================

Research Advance

### Comments on *'An airway organoid-based screen identifies a role for the HIF1α–glycolysis axis in SARS-CoV-2 infection'*

Xiaohua Duan[1,2], Hui Wang[2,*], David D. Ho[3,*], Robert E. Schwartz[4,5,*], Todd Evans[1,*], and Shuibing Chen[1,*]

[1] Department of Surgery, Weill Cornell Medicine, 1300 York Ave., New York, NY 10065, USA

[2] State Key Laboratory of Oncogenes and Related Genes, Center for Single-Cell Omics, School of Public Health, Shanghai Jiao Tong University School of Medicine, Shanghai 200025, China

[3] Aaron Diamond AIDS Research Center, Columbia University Vagelos College of Physicians and Surgeons, New York, NY 10032, USA

[4] Division of Gastroenterology and Hepatology, Department of Medicine, Weill Cornell Medicine, 1300 York Ave., New York, NY 10065, USA

[5] Department of Physiology, Biophysics and Systems Biology, Weill Cornell Medicine, 1300 York Ave., New York, NY 10065, USA

* Correspondence to: Shuibing Chen, E-mail: shc2034@med.cornell.edu; Todd Evans, E-mail: tre2003@med.cornell.edu; David D. Ho, E-mail: dh2994@cumc.columbia.edu; Robert E. Schwartz, E-mail: res2025@med.cornell.edu; Hui Wang, E-mail: huiwang@shsmu.edu.cn

Coronavirus disease 2019 (COVID-19) has been an ongoing public health crisis since the end of 2019; besides vaccine development there has been major research efforts focused on developing antiviral therapeutics. Remdesivir was the first FDA approved antiviral drug for COVID-19. Subsequently, the FDA granted emergency use authorization (EUA) for three monoclonal antibody treatments including sotrovimab or a combination of casirivimab and imdevimab, or bamlanivimab and etesevimab, each of which target the coronavirus spike protein to block viral entry. Most recently, Britain granted conditional authorization for the ribonucleoside analog Molnupiravir, developed by Merck as a viral replication inhibitor. The protease inhibitor PF-07321332 developed by Pfizer and boosted by ritonavir showed promising results in a Phase III clinical trial, reducing the risk of hospitalization or death by 89% compared to placebo.

ORIGINAL UNEDITED MANUSCRIPT

© The Author(s) 2021. Published by Oxford University Press on behalf of Institute of Biochemistry and Cell Biology, Shanghai Institutes for Biological Sciences, Chinese Academy of Sciences. This is an Open Access article distributed under the terms of the Creative Commons Attribution License (https://creativecommons.org/licenses/by/4.0/), which permits unrestricted reuse, distribution, and reproduction in any medium, provided the original work is properly cited.

Despite tremendous research efforts to combat the pandemic, there remains a need to better understand the viral life cycle and host response in disease relevant models. Cell-based models have been developed to study viral entry, life cycle, tropism, and pathogenesis. The African green monkey Vero E6 cell line expresses the ACE2 entry receptor and is commonly used to study virus entry and expansion (Hoffmann et al., 2020). The human cell lines HEK293T, Calu-3, Caco-2, and Huh7 are also permissive for viral infection *in vitro* (H. Chu et al., 2020). However, as these models are derived from human cancers or are quite different from their initial cell of origin they do not accurately mimic human physiological and pathological responses. Human primary cells (Hou et al., 2020) and adult organoids (Lamers et al., 2020; Salahudeen et al., 2020) may better model SARS-CoV-2 infection, but are limited by the scale for what is required for high-throughput drug screening. Organoids derived from human pluripotent stem cells (hPSCs) overcome such limitations, as they can be used to study infection of relevant normal human tissues and can be scaled for COVID-19 disease modeling and drug discovery (Yang et al., 2020; Duan et al., 2021; Han et al., 2021).

In the recent publication entitled ‘An airway organoid-based screen identifies a role for the HIF1α–glycolysis axis in SARS-CoV-2 infection’ (Duan et al., 2021), we developed a modified protocol to generate airway organoids from hPSCs (hPSC-AO). Single cell RNA sequencing (scRNA-seq) profiling showed that the organoids have a cellular composition similar to the adult human airway counterpart. Morphologically, hPSC-AOs contain beating cilia, a typical characteristic of adult airway tissue. Immunostaining and scRNA-seq data showed that ACE2 is expressed in acetyl-a-tubulin$^{+}$FOXJ1$^{+}$ ciliated-like cells. The hPSC-AOs are permissive to SARS-CoV-2 infection, and the ciliated cells are the main target, consistent with primary tissue data (Hou et al., 2020).

We then performed a high-content chemical screen using hPSC-AOs to identify anti-viral drugs. One compound, GW6471, was validated to decrease viral infection in a dose-dependent manner, independent of cytotoxicity. GW6471 also inhibited infection of hPSC-AOs by the B.1.351 SARS-CoV-2 variant, as well as inhibiting infection of hPSC-derived colon organoids. Therefore, GW6471 displays broad-spectrum anti-SARS-CoV-2 activity in multiple tissues.

Transcriptomic and metabolic profiling were applied to investigate the GW6471 mechanism of action. GW6471 was found to inhibit the Hypoxia Inducible Factor 1 subunit alpha (HIF1α) pathway when hPSC-AOs or hPSC-COs were treated after infection. A chemical inhibitor of HIF1α or short hairpin RNAs (shRNAs) targeting HIF1α both validated the essential role of HIF1α in permissiveness of SARS-CoV-2 infection. HIF1α is known as a classic upstream regulator of anaerobic glycolysis. Indeed, metabolic profiling identified decreased glycolysis following

ORIGINAL UNEDITED MANUSCRIPT

GW6471 treatment, consistent also with our transcriptomic profiling. Consistent with our results, a previous study of SARS-CoV-2 infected monocytes suggested that enhanced glycolysis induced by increased HIF1α levels can sustain viral replication (Codo et al., 2020). The higher rate of glycolysis leads to decreased pyruvate metabolism in the mitochondria, which is the rate-limiting intermediate metabolite in the conversion of carbohydrates into fatty acids and cholesterol. Our metabolic profiling data also shows a lower level of fatty acid synthesis in the GW6471 treatment group. Finally, three compounds targeting key steps of fatty acid synthesis, including xanthohumol (an inhibitor of diacylglycerol acetyltransferase), 5-(tetradecyloxy)-2-furoic acid (TOFA) and ND-646 (both inhibitors of acetyl-coA carboxylase), were confirmed to block SARS-CoV-2 infection. A recent study supports our conclusion, that blocking fatty acid synthesis reduces SARS-CoV-2 infection (J. Chu et al., 2021). Together, transcriptomic and metabolic profiling revealed a key role for the HIF1α–glycolysis–fatty acid synthesis axis in mediating productive SARS-CoV-2 infection.

In summary, our study presents a hPSC-AO based model to study the interactions between host human tissues and SARS-CoV-2. By performing a high content chemical screen and subsequent mechanistic studies, we identified a critical role for the HIF1α–glycolysis–fatty acid synthesis axis during SARS-CoV-2 infection, which contains several druggable targets for anti-SARS-CoV-2 drug development. Further studies are needed to develop targeting of the host metabolic state into a clinical therapeutic strategy (Figure 1).

### References

Chu, H., Chan, J.F., Yuen, T.T., et al. (2020). Comparative tropism, replication kinetics, and cell damage profiling of SARS-CoV-2 and SARS-CoV with implications for clinical manifestations, transmissibility, and laboratory studies of COVID-19: an observational study. Lancet Microbe *1*, e14-e23.

Chu, J., Xing, C., Du, Y., et al. (2021). Pharmacological inhibition of fatty acid synthesis blocks SARS-CoV-2 replication. Nat Metab.

Codo, A.C., Davanzo, G.G., Monteiro, L.B., et al. (2020). Elevated Glucose Levels Favor SARS-CoV-2 Infection and Monocyte Response through a HIF-1α/Glycolysis-Dependent Axis. Cell Metab *32*, 437-446 e435.

Duan, X., Tang, X., Nair, M.S., et al. (2021). An airway organoid-based screen identifies a role for the HIF1α–glycolysis axis in SARS-CoV-2 infection. Cell Rep *37*, 109920.

Han, Y., Duan, X., Yang, L., et al. (2021). Identification of SARS-CoV-2 inhibitors using lung and colonic organoids. Nature *589*, 270-275.

Hoffmann, M., Kleine-Weber, H., Schroeder, S., et al. (2020). SARS-CoV-2 Cell Entry Depends on ACE2 and TMPRSS2 and Is Blocked by a Clinically Proven Protease Inhibitor. Cell *181*, 271-280.e278.

Hou, Y.J., Okuda, K., Edwards, C.E., et al. (2020). SARS-CoV-2 Reverse Genetics Reveals a Variable Infection Gradient in the Respiratory Tract. Cell *182*, 429-446 e414.

Lamers, M.M., Beumer, J., van der Vaart, J., et al. (2020). SARS-CoV-2 productively infects human gut enterocytes. Science *369*, 50-54.

Salahudeen, A.A., Choi, S.S., Rustagi, A., et al. (2020). Progenitor identification and SARS-CoV-2 infection in human distal lung organoids. Nature *588*, 670-675.

ORIGINAL UNEDITED MANUSCRIPT

Yang, L., Han, Y., Nilsson-Payant, B.E., et al. (2020). A Human Pluripotent Stem Cell-based Platform to Study SARS-CoV-2 Tropism and Model Virus Infection in Human Cells and Organoids. Cell Stem Cell *27*, 125-136 e127.

ORIGINAL UNEDITED MANUSCRIPT

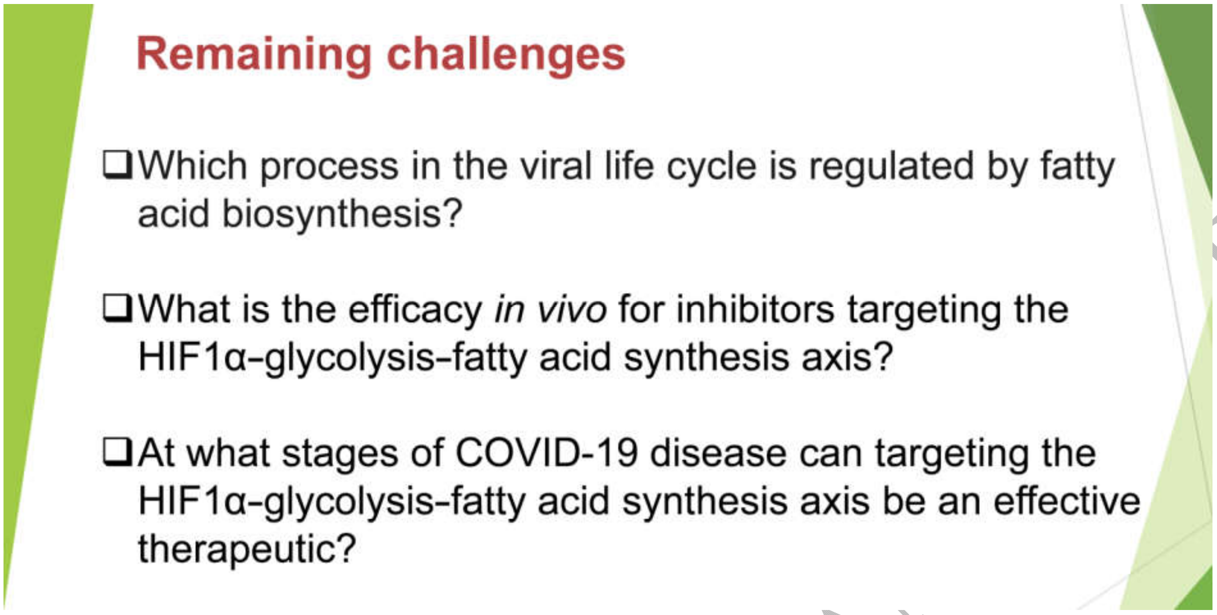

**Figure 1** Remaining challenges for the development of therapeutics to inhibit SARS-CoV-2 infection through targeting the HIF1α–glycolysis–fatty acid synthesis axis.

ORIGINAL UNEDITED MA[illegible]T
